# Ferulic Acid Is a Nutraceutical β-Secretase Modulator That Improves Behavioral Impairment and Alzheimer-like Pathology in Transgenic Mice

**DOI:** 10.1371/journal.pone.0055774

**Published:** 2013-02-08

**Authors:** Takashi Mori, Naoki Koyama, Marie-Victoire Guillot-Sestier, Jun Tan, Terrence Town

**Affiliations:** 1 Department of Biomedical Sciences, Saitama Medical Center and University, Kawagoe, Saitama, Japan; 2 Department of Pathology, Saitama Medical Center and University, Kawagoe, Saitama, Japan; 3 Department of Biomedical Sciences, Cedars-Sinai Medical Center, Los Angeles, California, United States of America; 4 Rashid Laboratory for Developmental Neurobiology, Silver Child Development Center, Department of Psychiatry and Behavioral Neurosciences, Morsoni College of Medicine, University of South Florida, Tampa, Florida, United States of America; 5 Neuroimmunology Laboratory, Department of Psychiatry and Behavioral Neurosciences, Morsoni College of Medicine, University of South Florida, Tampa, Florida, United States of America; 6 Regenerative Medicine Institute Neural Program, Cedars-Sinai Medical Center, Los Angeles, California, United States of America; 7 Department of Medicine, David Geffen School of Medicine, University of California, Los Angeles, Los Angeles, California, United States of America; Boston University School of Medicine, United States of America

## Abstract

Amyloid precursor protein (APP) proteolysis is required for production of amyloid-β (Aβ) peptides that comprise β-amyloid plaques in brains of Alzheimer’s disease (AD) patients. Recent AD therapeutic interest has been directed toward a group of anti-amyloidogenic compounds extracted from plants. We orally administered the brain penetrant, small molecule phenolic compound ferulic acid (FA) to the transgenic PSAPP mouse model of cerebral amyloidosis (bearing mutant human APP and presenilin-1 transgenes) and evaluated behavioral impairment and AD-like pathology. Oral FA treatment for 6 months reversed transgene-associated behavioral deficits including defective: hyperactivity, object recognition, and spatial working and reference memory, but did not alter wild-type mouse behavior. Furthermore, brain parenchymal and cerebral vascular β-amyloid deposits as well as abundance of various Aβ species including oligomers were decreased in FA-treated PSAPP mice. These effects occurred with decreased cleavage of the β-carboxyl-terminal APP fragment, reduced β-site APP cleaving enzyme 1 protein stability and activity, attenuated neuroinflammation, and stabilized oxidative stress. As *in vitro* validation, we treated well-characterized mutant human APP-overexpressing murine neuron-like cells with FA and found significantly decreased Aβ production and reduced amyloidogenic APP proteolysis. Collectively, these results highlight that FA is a β-secretase modulator with therapeutic potential against AD.

## Introduction

Alzheimer’s disease (AD) represents a worldwide public health threat, and is characterized by progressive dementia ultimately leading to death [1]. Based on the “amyloid cascade hypothesis” of AD, which purports that cerebral amyloid-β (Aβ) peptide accumulation sets a neurotoxic cascade into motion [2]–[4], a great deal of focus has been directed toward anti-amyloid therapies that reduce production or enhance clearance of cerebral Aβ [5]–[10]. Although considerable effort has been made in this area, designer drugs for treatment of AD patients have not yet panned out in the clinic. Indeed, increasing numbers of agents have been abandoned worldwide by pharmaceutical companies, largely due to toxicity and efficacy issues in pre-clinical rodent models and in clinical trials. Furthermore, current standard-of-care pharmacotherapeutics (i.e., acetylcholinesterase inhibitors or N-methyl D-aspartate antagonists) show only modest symptomatic benefit, especially when administered in advanced stages of the disease. Currently, a number of clinical trials are underway based on the amyloid cascade hypothesis, and we await results from those studies.

Additionally, synthetic compounds can have undesirable side effects, especially when given long-term in a disease prevention paradigm. For example, the ADAPT trial to test non-steroidal anti-inflammatory drugs for primary AD prevention failed to complete due to non-steroidal anti-inflammatory drug-associated cardiotoxicity [11], [12]. On the other hand, naturally occurring dietary compounds, or “nutraceuticals”, typically have fewer side effects than designer drugs [13]. Amongst these, plant-derived compounds represent an alternative class of therapeutic tools for a variety of diseases including neurodegenerative disorders, cancer, diabetes, cardiovascular disease, inflammatory diseases, and even aging [14].

**Figure 1:**
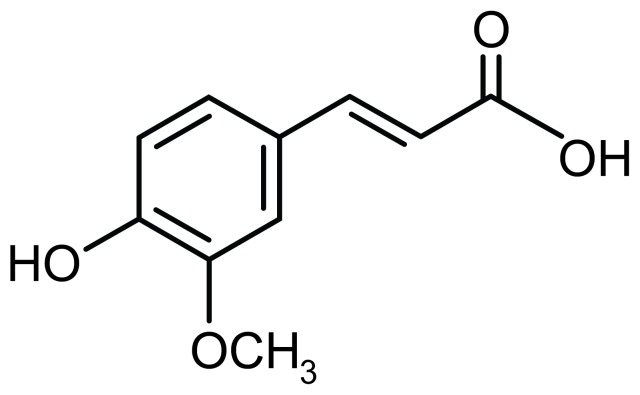
Chemical structure of ferulic acid (FA: C_10_H_10_O_4_). FA (*trans*-4-hydroxy-3-methoxycinnamic acid) is a phenolic compound.

Ferulic acid (*trans*-4-hydroxy-3-methoxycinnamic acid; FA) (Fig. 1) is one of the most abundant phenolic compounds in the human diet. FA is found in seed plants (e.g., rice, wheat, and oat), vegetables (e.g., tomato and carrot), and fruits (e.g., pineapple and orange), and has free radical scavenging and antioxidant properties [15]. In the context of neurological disease, intravenous administration of FA has been shown to protect against neuronal cell death induced by cerebral ischemia [16]–[18]. Interestingly, FA is also known to promote neural progenitor cell proliferation *in vitro* and *in vivo*, which has been demonstrated to ameliorate stress-induced depression-like behavior in mice [19]. Collectively, these results led us to test whether FA might attenuate AD-like pathology in a transgenic mouse model of cerebral amyloidosis.

To evaluate this hypothesis, we orally administered the compound to an accelerated mouse model of cerebral amyloidosis [bearing amyloid precursor protein (APP) “Swedish” APP_K595N/M596L_ (APPswe) and Presenilin 1 (PS1) exon 9 deleted (PS1_ΔE9_) mutant human transgenes; designated PSAPP mice] for 6 months, commencing at 6 months of age, and evaluated behavioral impairment, AD-like pathology, APP processing, neuroinflammation, and oxidative stress responses at 12 months of age. Complementary analyses were conducted *in vitro* utilizing mutant human APP-overexpressing murine N2a neuron-like cells.

## Results

### Remediation of Behavioral Impairment in Ferulic Acid-treated PSAPP Mice

**Figure 2:**
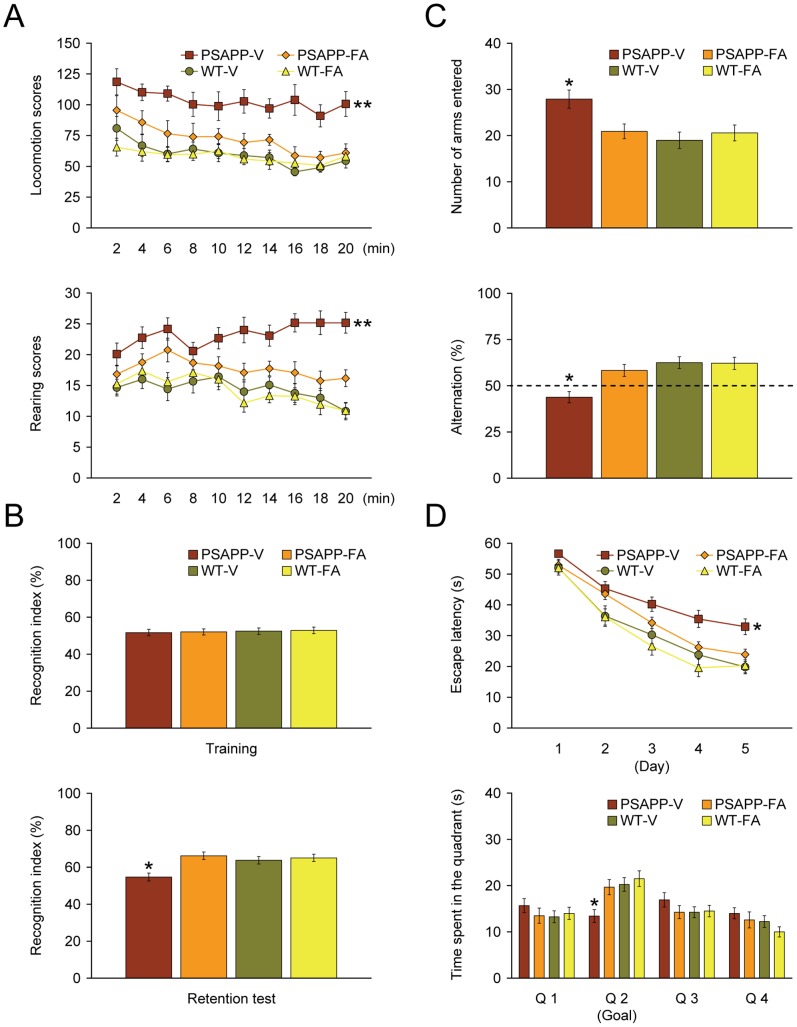
Complete remediation of behavioral impairment in ferulic acid (FA)-treated PSAPP mice. Data were obtained from PSAPP mice treated with vehicle (PSAPP-V, n = 12) or with FA (PSAPP-FA, n = 12) and also wild-type mice treated with vehicle (WT-V, n = 12) or with FA (WT-FA, n = 12) for 6 months beginning at 6 months of age, and subjected to behavioral testing at 12 months of age. (A) Locomotion (upper panel) and rearing (lower panel) scores obtained from open field activity testing are shown. (B) Recognition index (%) in the object recognition test is shown from training (upper panel) and retention test phases (lower panel). (C) Y-maze test data are represented as total arm entry (upper panel) and spontaneous alternation (lower panel) measures. (D) Morris water maze data are shown from the submerged platform test (upper panel, learning phase) and from a single 60-second probe trial test (lower panel), conducted 1 day after termination of the learning phase. All statistical comparisons are vs. PSAPP-V mice.

We started by orally administering FA or vehicle to PSAPP or wild-type (WT) mice starting at 6 months of age, for a period of 6 months. Baseline cognitive status was determined in untreated PSAPP vs. WT mice at 6 months of age just prior to dosing, but did not show cognitive impairment in a comprehensive testing battery (data not shown). Behavioral assessment was also made at the conclusion of treatment (12 months of age). When placed into a novel environment, PSAPP-V mice exhibited hyperactivity as measured by higher locomotion and rearing scores compared with the other 3 groups of mice (Fig. 2A). This behavioral phenotype has been noted in mouse models of cerebral amyloidosis (e.g., Tg2576 or PSAPP mice) [9], [20]–[22], and may reflect disinhibition resulting from cortical and/or hippocampal injury [23]. Overall analysis of variance (ANOVA) showed main effects of time (p = 0.001 for locomotion scores), genotype (p<0.001 for both locomotion and rearing scores), and treatment (p<0.001 for both locomotion and rearing scores), and repeated-measures ANOVA followed by *post hoc* comparisons revealed statistically significant differences between PSAPP-V mice and the other 3 mouse groups for both locomotion and rearing scores (Fig. 2A, **p<0.01 for PSAPP-V vs. PSAPP-FA, WT-V, or WT-FA mice). Hyperactivity was fully prevented in PSAPP-FA mice, as they did not statistically differ from WT-V or WT-TA animals (p>0.05).

We then tested learning and memory by novel object recognition test in the same cohort of mice. If mice remember an initial encounter with a novel object, they tend to preferentially explore the new vs. familiar object, typically operationalized as “recognition index” [24]. Although all groups performed similarly during the training phase of the test, in the retention phase, one-way ANOVA followed by *post hoc* comparison showed statistically significant differences on recognition index between PSAPP-V mice and the other 3 mouse groups (Fig. 2B, *p<0.05 for PSAPP-V vs. PSAPP-FA, WT-V, or WT-FA mice). Of note, PSAPP-FA mice had significantly increased novel object exploration frequency vs. PSAPP-V animals (Fig. 2B), but did not significantly differ from WT-V or WT-FA groups (p>0.05), showing that FA improved novel object recognition impairment associated with PSAPP transgene expression.

Exploratory activity and spatial working memory were further examined by Y-maze total arm entry and spontaneous alternation tests. One-way ANOVA followed by *post hoc* comparison disclosed statistically significant differences on Y-maze total arm entries between PSAPP-V mice and the other 3 mouse groups (Fig. 2C, *p<0.05 for PSAPP-V vs. PSAPP-FA, WT-V, or WT-FA mice). This index of hyperactivity was totally alleviated in PSAPP-FA mice, as they did not statistically differ from WT-V or WT-FA animals (p>0.05).

Mice tend to spontaneously alternate arm entries in the Y-maze, such that they will visit the three arms in sequence more frequently than would occur by chance (50%, see dotted line in Fig. 2C); this is generally interpreted as a measure of spatial working memory. One-way ANOVA followed by *post hoc* testing showed statistically significant differences on Y-maze spontaneous alternation between PSAPP-V mice and the other 3 mouse groups (Fig. 2C, *p<0.05 for PSAPP-V vs. PSAPP-FA, WT-V, or WT-FA mice). Importantly, PSAPP-V mice had less tendency to alternate, whereas PSAPP-FA animals had significantly increased alternation behavior (Fig. 2C), but did not significantly differ from WT-V or WT-FA groups (p>0.05), demonstrating that oral FA treatment completely improved defective spatial working memory in PSAPP mice.

The same cohort of animals was further evaluated in the Morris water maze, a widely accepted assay of spatial reference learning and memory in rodents [25], [26]. For the learning phase of the test, overall ANOVA showed main effects of time (p<0.001), treatment (p<0.05), and genotype (p<0.001), and repeated-measures ANOVA followed by *post hoc* comparison revealed statistically significant differences between PSAPP-V mice and the other 3 mouse groups (Fig. 2D, *p<0.05 for PSAPP-V vs. PSAPP-TA, WT-V, or WT-TA mice). PSAPP-V mice had longer latency to reach the platform location after training than the other 3 mouse groups, whereas PSAPP-FA mice had significantly shorter latencies, indicating improvement. For the probe trial (day 6 of testing), the invisible platform was removed from the pool and platform location memory was evaluated. When considering quadrant 2 (Q 2, goal quadrant) data, one-way ANOVA followed by *post hoc* testing revealed statistically significant differences between PSAPP-V mice and the other 3 mouse groups (Fig. 2D, *p<0.05 for PSAPP-V vs. PSAPP-FA, WT-V, or WT-FA mice). PSAPP-FA mice swam in the goal quadrant significant longer than PSAPP-V mice, and their behavior did not significantly differ from WT-V or WT-FA mice, demonstrating that 6-month FA treatment completely remediated PSAPP transgene-associated spatial memory impairment.

We ruled out the possibility that behavioral differences in the Morris water maze were due to motivational issues or to locomotor impairment, as there were no significant between-groups differences (p>0.05) on swim speed during either the learning or probe trial phases of the test. Importantly, degree of thigmotaxis could indicate levels of anxiety and impact interpretation of Morris water maze results. However, we did not observe evidence of thigmotaxis, operationalized as prolonged movement of the mice along the pool circumference, in any animals examined during either the learning or probe trial phases of the test. Finally, for all of the behavioral tests conducted, we used multiple ANOVA models with gender as a categorical covariate, but did not detect significant gender main effects or interactive terms (p>0.05). We also stratified by gender and found a similar pattern of results as above in both males and females (data not shown).

### Amelioration of Alzheimer-like Pathology in an Accelerated Mouse Model of Cerebral Amyloidosis

**Figure 3:**
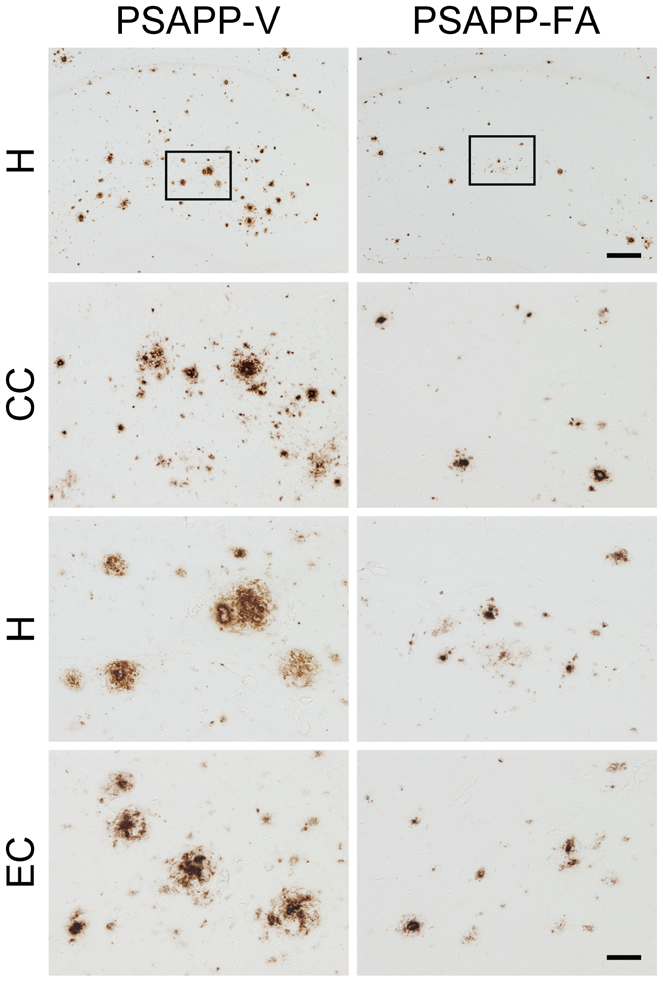
Cerebral parenchymal β-amyloid deposits are markedly attenuated in PSAPP mice after oral ferulic acid (FA) treatment. Representative photomicrographs were obtained from PSAPP mice treated with vehicle (PSAPP-V) or with FA (PSAPP-FA) for 6 months starting at 6 months of age (mouse age at sacrifice = 12 months). 4G8 immunohistochemistry is depicted, revealing cerebral β-amyloid deposits in PSAPP-V and PSAPP-TA mice. Brain regions shown include: cingulate cortex (CC, top), hippocampus (H, middle), and entorhinal cortex (EC, bottom). Middle H panels are higher magnification images from insets in the upper H panels. Scale bars denote 250 µm (upper) and 50 µm (lower).

To evaluate if FA treatment altered Aβ/β-amyloid pathology, we performed 1) conventional β-amyloid “burden” analysis using a monoclonal antibody against Aβ_17–24_ (4G8), 2) β-amyloid plaque morphometric analysis, and 3) separate Aβ_1–40_ and Aβ_1–42_ sandwich enzyme-linked immunosorbent assays (ELISAs). PSAPP-V mice had typical β-amyloid deposition at 12 months of age that distributed diffusely throughout cingulate cortex (CC), entorhinal cortex (EC), and hippocampus (H) regions [22], [27], [28], and was markedly and significantly reduced by 59 to 63% in CC, EC, and H regions of PSAPP-FA mouse brains in absence of sub-region specific effects (Fig. 3, 4A, ***p<0.001). It is noteworthy that PSAPP mice at 6 months of age (when dosing started), had quantitatively minor (0.5 to 0.7%) cerebral β-amyloid burden, and the majority of these deposits were seed-like dots <25 µm in size, with only few deposits between 25 and 50 µm (data not shown). Thus, while 6-month treatment with FA reduced cerebral amyloid deposition kinetics, it was not able to completely prevent this pathological feature (Fig. 3, 4A). Importantly, FA reduction of β-amyloid deposits was independent of gender (data not shown).

**Figure 4:**
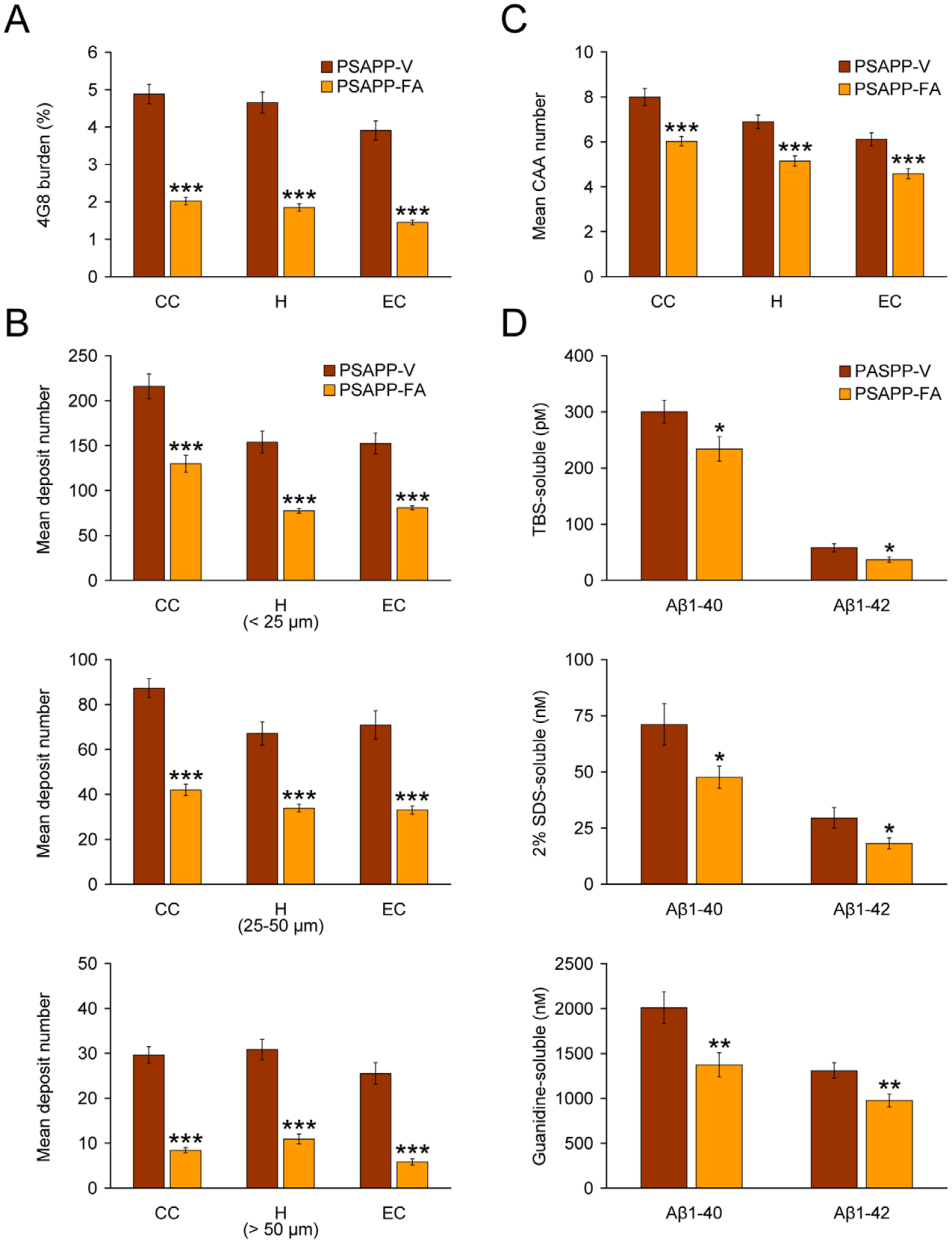
Oral ferulic acid (FA) treatment reduces cerebral parenchymal and vascular β-amyloid deposits and brain amyloid-β (Aβ) levels in PSAPP mice. Data were obtained from PSAPP mice treated with vehicle (PSAPP-V, n = 12) or with FA (PSAPP-FA, n = 12) for 6 months commencing at 6 months of age (mouse age at sacrifice = 12 months). (A) Quantitative image analysis for Aβ (4G8) burden is shown, and each brain region is indicated on the x-axis [cingulate cortex (CC), hippocampus (H), and entorhinal cortex (EC)]. (B) Morphometric analysis of cerebral parenchymal β-amyloid deposits is shown in PSAPP-V and PSAPP-FA mice. Brain coronal sections were stained with 4G8 antibody, and deposits were blindly counted based on maximum diameter and assigned to one of three mutually exclusive categories: small (<25 µm; top), medium (between 25 and 50 µm; middle), or large (>50 µm; bottom). Mean plaque subset number per mouse is shown on the y-axis, and each brain region is represented on the x-axis. (C) Severity of cerebral amyloid angiopathy (mean CAA deposit number per mouse) is shown on the y-axis with brain region indicated on the x-axis. (D) TBS-soluble, 2% SDS-soluble, and TBS-insoluble (but 5M guanidine HCl-extractable) fractions from three-step extracted brain homogenates were separately measured by sandwich ELISA for human Aβ_1–40_ and Aβ_1–42_. Statistical comparisons for A–D are within each brain region and/or Aβ species, and between PSAPP-V and PSAPP-FA mice.

To assess whether reduced β-amyloid burden was specific to a particular plaque size subset or occurred more generally, morphometric analysis of β-amyloid deposits was performed in both FA- and vehicle-treated PSAPP mice. According to previously described methods [7], [9], [22], [29], [30], β-amyloid deposits were assigned to one of three mutually exclusive categories according to maximum diameter: small (<25 µm), medium (between 25 and 50 µm), or large (>50 µm). Mean numbers of deposits in all three subsets showed statistically significant decreases in PSAPP-FA vs. PSAPP-V mice across all three brain regions examined, with greatest reduction in the large-sized subset (Fig. 3, 4B, ***p<0.001, % reduction for: small, 40 to 50%; medium, 49 to 53%; large, 65 to 77%). Stratification by gender revealed the same pattern of results in both males and females (data not shown).

In addition to brain parenchymal deposition of β-amyloid as senile plaques, 83% of AD patients manifest cerebral vascular β-amyloid deposits, known as cerebral amyloid angiopathy (CAA) [31]. PSAPP mice also develop vascular β-amyloid deposits with age [22], [28]. In PSAPP-V mice, cerebral vascular β-amyloid deposits were frequently observed in walls of penetrating arteries at the pial surface in CC and EC regions and in small arteries at the hippocampal fissure. We scored Aβ antibody (4G8)-stained cerebral vascular deposits in PSAPP-V and PSAPP-FA mice and found significant reductions in PSAPP-FA mouse brains in all three brain regions examined (Fig. 4C, ***p<0.001).

In support of the above observations, biochemical analyses of Aβ species in brain homogenates from PSAPP-FA mice revealed statistically significant reductions in both Aβ_1–40_ and Aβ_1–42_ abundance in the TBS-soluble and detergent-soluble fractions (Fig. 4D, 22 to 39%; *p<0.05). Moreover, the guanidine-HCl-soluble fraction, which most closely reflects Aβ deposits detected by immunohistochemistry, disclosed statistically significant reductions in PSAPP-FA mice for both Aβ_1–40_ and Aβ_1–42_ abundance (Fig. 4D, 25 to 32%; **p<0.01). Together, these data show that FA delays progression of cerebral amyloidosis including brain parenchymal and cerebral vascular β-amyloid deposits as well as Aβ_1–40_ and Aβ_1–42_ abundance.

### Modulation of β-secretase in PSAPP Mouse Brains Treated with Ferulic Acid

**Figure 5:**
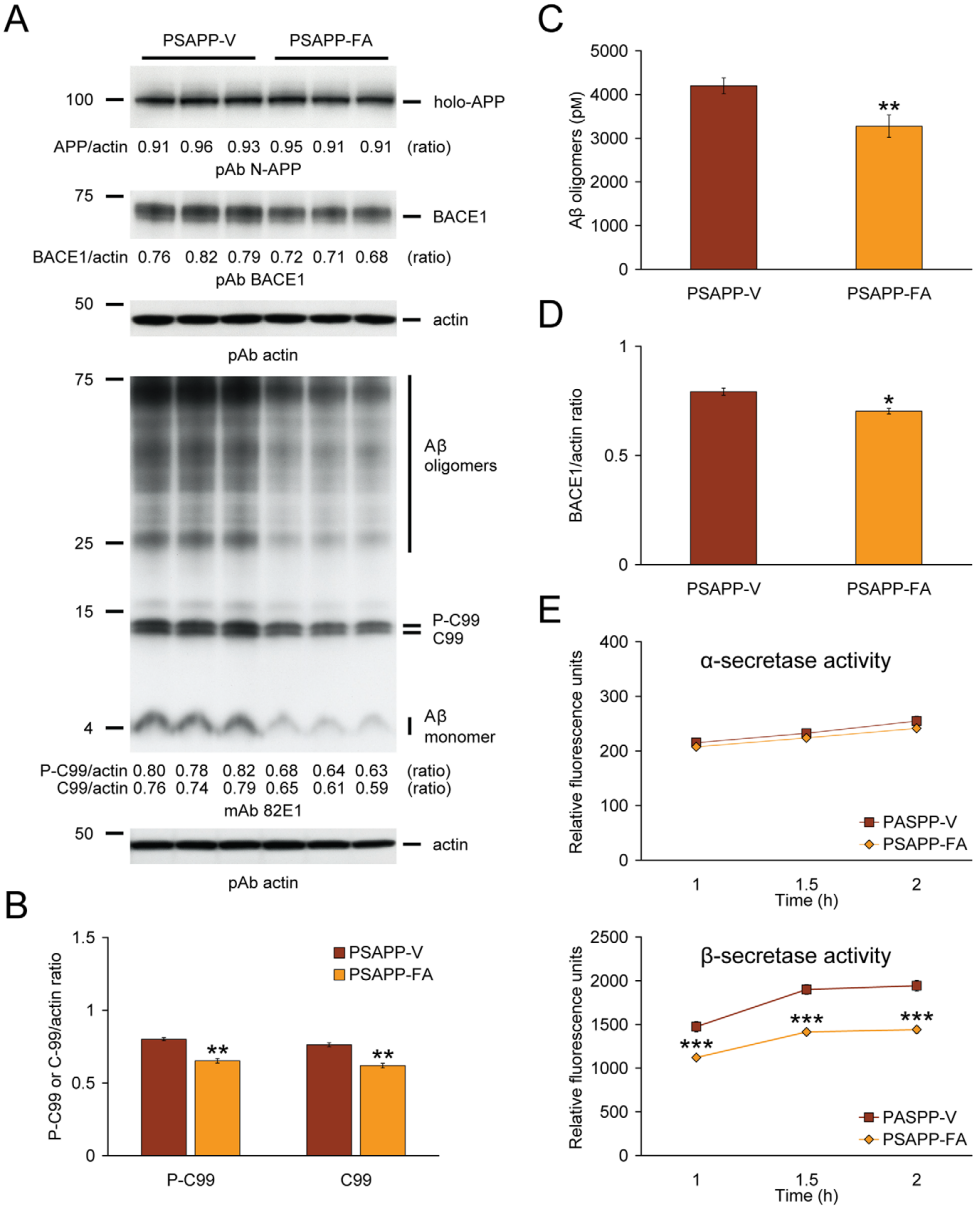
β-site amyloid precursor protein (APP) cleaving enzyme 1 (BACE1) modulation and inhibition of amyloidogenic APP processing in PSAPP mice treated with ferulic acid (FA). (A) Western blots are shown using an amino (N)-terminal APP polyclonal antibody (pAb N-APP; holo-APP is shown) and a carboxyl-terminal BACE1 polyclonal antibody (pAb BACE1). Western blots are also shown using an amino-terminal amyloid-β (Aβ) monoclonal antibody (mAb 82E1), which detects various amyloidogenic APP cleavage fragments including: Aβ monomer and oligomers as well as phospho-C99 (P-C99) and non-phospho-C99 (C99). Actin is shown as a loading control for each blot, and ratiometric densitometry data are shown below each lane. (B) Densitometry analyses are shown for ratios of C-99 or P-C99 to actin. (C) Aβ oligomers in the 2% SDS-soluble brain homogenate fraction were measured by sandwich ELISA. (D) Densitometry analysis is shown for ratio of BACE1 to actin. (E) α- and β-secretase activity assays are shown. Relative fluorescence units are depicted on the y-axis, and reaction time is represented on the x-axis. Representative Western blots for A were obtained from PSAPP mice treated with vehicle (PSAPP-V, n = 3) or with FA (PSAPP-FA, n = 3). Data for B-E were obtained from PSAPP mice treated with vehicle (PSAPP-V, n = 12) or with FA (PSAPP-FA, n = 12) for 6 months beginning at 6 months of age. All statistical comparisons are between PSAPP-V and PSAPP-FA mice.

We reasoned that ameliorated cerebral amyloidosis in PSAPP-FA mouse brains could be due to three possibilities: 1) increased brain-to-blood Aβ efflux [32], 2) decreased expression of APP or PS1 transgenes, or 3) inhibition of amyloidogenic APP processing. We started by analyzing peripheral blood samples from PSAPP-V and PSAPP-FA mice at the time of sacrifice for plasma Aβ_1–40_ and Aβ_1–42_ species, but did not detect between-groups differences (data not shown). To rule out the possibility that attenuated cerebral amyloidosis in FA-treated PSAPP mouse brains was due to decreased expression of transgene-derived APP or PS1, brain homogenates from PSAPP-V and PSAPP-FA mice were probed using amino-terminal APP polyclonal or carboxyl-terminal PS1 monoclonal antibodies, and comparable band intensities were noted for APP or PS1 holoprotein levels (Fig. 5A, data not shown).

Given these null results, we shifted our attention to examining the third possibility, reduced amyloidogenic APP metabolism. Thus, brain homogenates from PSAPP-FA and PSAPP-V mice were probed with an amino-terminal Aβ_1–16_ monoclonal antibody (82E1) that reacts with both amyloidogenic β-C terminal APP fragment (CTF, C99) and phospho-β-CTF (P-C99), as well as monomeric and oligomeric Aβ species. Densitometry confirmed that APP metabolism to C99 and P-C99 was significantly deceased in PSAPP-FA mice (Fig. 5A, B, **p<0.01). These effects were accompanied by reduced abundance of Aβ species between 25 to 75 kDa (presumed Aβ oligomers) and monomeric Aβ in PSAPP-FA mice (Fig. 5A). In order to verify attenuated expression of Aβ oligomers in FA-treated brain homogenates by Western blot analysis, quantitation was performed by sandwich ELISA in the detergent-soluble fraction from PSAPP-FA and PSAPP-V mice. Data revealed significant reductions in PSAPP-FA vs. PSAPP-V mice (Fig. 5C, **p<0.01).

We next aimed to mechanistically probe FA suppression of Aβ levels *in vivo*. Given decreased Aβ, C-99, and P-C99 abundance in response to FA treatment, we hypothesized that FA could modulate β-secretase activity, either directly or indirectly. β-site APP cleaving enzyme 1 (BACE1) is a type I transmembrane aspartyl protease predominately responsible for processing APP into soluble APP-β (sAPP-β) and the amyloidogenic CTF, C99. C99 is then cleaved by the γ-secretase complex, releasing Aβ species of various lengths [33]–[37]. To investigate whether long-term FA treatment modulated β-secretase, brain homogenates were probed with a carboxyl-terminal BACE1 polyclonal antibody. Densitometry showed modestly but significantly decreased BACE1 protein abundance in PSAPP-FA mouse brain homogenates (Fig. 5A, D, *p<0.05). To further verify whether FA altered BACE1 expression at the transcriptional level, relative expression levels of BACE1 mRNA were assayed in mouse brain homogenates by quantitative real-time PCR (QRT-PCR), but no significant between-groups differences were found (expressed as mean relative fold over WT-V mice ±1 S.E.; PSAPP-V mice: 0.96±0.06, PSAPP-FA mice: 0.94±0.06, WT-FA mice: 1.02±0.07). When taken together, these data suggested that FA operates at the protein level to destabilize BACE1, and led us to test whether FA altered its enzymatic activity. Results showed significantly attenuated β-secretase activity (but unaltered α-secretase activity) in brain homogenates from PSAPP-FA vs. PSAPP-V mice (Fig. 5E, ***p<0.001 at each time-point).

### Reduced Aβ Production and Inhibited Amyloidogenic APP Metabolism in Neuron-like Cells

**Figure 6:**
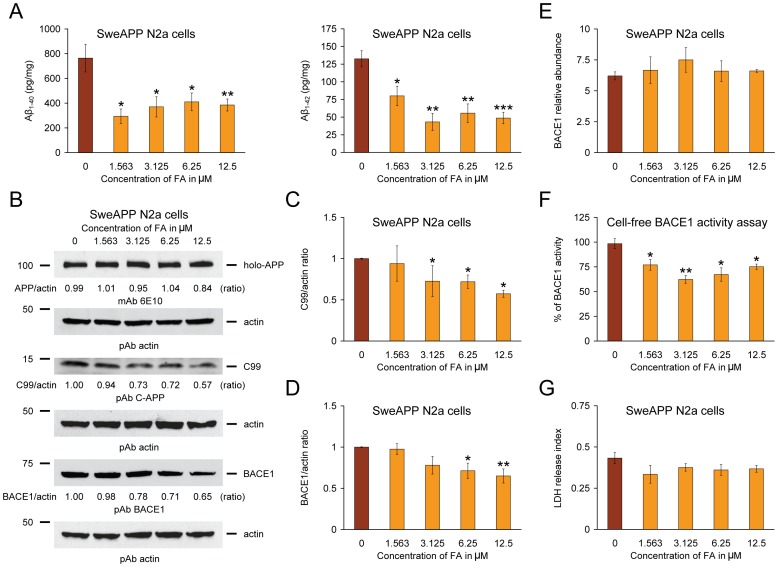
Ferulic acid (FA) inhibits amyloidogenic amyloid precursor protein (APP) metabolism in SweAPP N2a cells by modulating β-site APP cleaving enzyme 1 (BACE1) expression and activity. (A) Amyloid-β (Aβ)_1–40_ and Aβ_1–42_ species were separately measured in cell supernatants from SweAPP N2a cells by sandwich ELISAs. (B) Inhibition of amyloidogenic APP processing in SweAPP N2a cells treated with various doses of FA. Western blots using an amino-terminal Aβ_1–17_ monoclonal antibody (mAb 6E10), a carboxyl-terminal APP polyclonal antibody (pAb C-APP), or a carboxyl-terminal BACE1 polyclonal antibody (pAb BACE1) show holo-APP, carboxyl-terminal fragment generated by amyloidogenic APP cleavage (C99, β-CTF), and BACE1, respectively. Actin is included as an internal reference control, and ratiometric densitometry data are shown below each lane. (C) Densitometry results are shown as ratio of C99 to actin at various FA treatment doses. (D) Densitometry results are shown as ratio of BACE1 to actin at various FA treatment doses. (E) Quantitative real-time PCR results for BACE1 mRNA levels at various FA treatment doses are shown in arbitrary units, and BACE1 relative abundance is depicted on the y-axis. (F) Cell-free BACE1 activity assay results are displayed, and % of BACE1 activity is shown on the y-axis. (G) Lactate dehydrogenase (LDH) release assay results are shown for SweAPP N2a cells treated with 0 to 12.5 µM of FA. All statistical comparisons are vs. 0 µM of FA, and similar results were observed in 3–4 independent experiments.

Mitigated cerebral amyloidosis and polarized amyloidogenic APP metabolism in PSAPP-FA mice could be due to a direct, neuron cell autonomous affect or to an indirect mode of FA action. To determine whether FA could directly modulate APP metabolism in neuron-like cells, N2a cells that stably overexpresses human “Swedish”-mutated APP-695 (SweAPP N2a cells) were treated with a dose-range of FA. As shown in Fig. 6A, FA inhibited both Aβ_1–40_ and Aβ_1–42_ release into the media by separate sandwich ELISAs. Significant reduction for both Aβ species was evident even at the lowest dose (1.563 µM) of FA [*p<0.05; **p<0.01; ***p<0.001 for each dose vs. phosphate-buffered saline (PBS) control].

To investigate whether these effects were due to reduced amyloidogenic APP metabolism, Western blots were performed with a carboxyl-terminal APP polyclonal antibody that detects amyloidogenic C99 (Fig. 6B). Results qualitatively showed less abundance of C99 with increasing doses of FA, whereas holo-APP expression remained unaffected by FA treatment. Significant differences were found when comparing PBS-treated SweAPP N2a cells (control) to cells that were challenged with 3.125 to 12.5 µM of FA (Fig. 6C, *p<0.05). To determine whether BACE1 protein levels were reduced by FA treatment, SweAPP N2a cell lysates were probed with a carboxyl-terminal BACE1 polyclonal antibody. Significant differences were found when comparing PBS-treated (control) SweAPP N2a cells to cells that were treated with 6.25 to 12.5 µM of FA (Fig. 6D, *p<0.05; **p<0.01).

To assess *in vitro* whether FA targets BACE1 at transcription level, relative abundance of BACE1 mRNA was assayed in lysates from SweAPP N2a cells treated with various doses of FA (1.563 to 12.5 µm) or PBS control by QRT-PCR analysis. No significant differences were detected between-groups (Fig. 6E), suggesting a post-transcriptional mode of FA action on BACE1. Consistent with BACE1 *in vivo* data, these results further suggest that FA operates on BACE1 at the protein level.

To determine whether FA directly or indirectly inhibited BACE1 activity, we developed a BACE1 activity assay consisting of combining a dose range of FA in a cell-free system with BACE1 enzyme and fluorogenic reporters. The result was positive, as one-way ANOVA revealed a significant main effect of FA dose (p<0.005) and *post hoc* testing showed significant reductions from 1.563 to 12.5 µM of FA vs. 100% BACE1 activity (fluorescent emission of the substrate incubated with BACE1 enzyme alone) (Fig. 6F, *p<0.05; **p<0.01). Of note, significant reduction for BACE1 activity was evident even at the lowest dose (1.563 µM) of FA used (Fig. 6F, *p<0.05). As a positive control, BACE1 inhibitor II treatment (IC_50_ = 0.97 µM) at 1.25 µM revealed an inhibitory effect for BACE1 (53.1±6.8% of 100% BACE1 activity, **p<0.01).

Finally, to determine whether the above effects of FA might be attributable to cellular toxicity, SweAPP N2a cells were challenged with escalating doses of FA prior to lactate dehydrogenase release cytotoxicity assay. However, FA toxicity was not observed (Fig. 6G). When taken together, these data show that FA reduces β-secretase cleavage of APP and consequent amyloidogenic APP metabolism in neuron-like cells.

### Reduced Neuroinflammation and Oxidative Stress in PSAPP Mice

**Figure 7:**
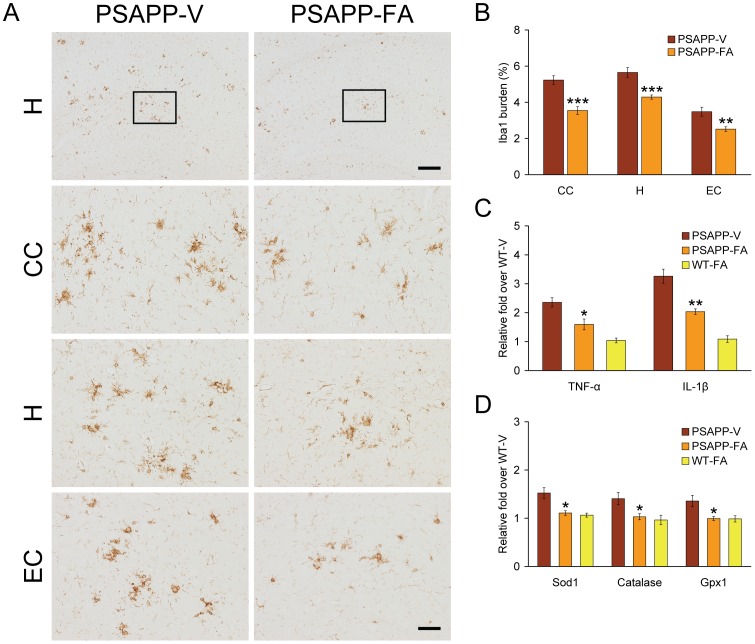
Neuroinflammation and oxidative stress markers are attenuated in ferulic acid (FA)-treated PSAPP mice. (A-B) Data were obtained from PSAPP mice treated with vehicle (PSAPP-V, n = 12) or with FA (PSAPP-FA, n = 12) for 6 months commencing at 6 months of age. (C-D) Data were obtained from PSAPP mice treated with vehicle (PSAPP-V, n = 12) or with FA (PSAPP-FA, n = 12) as well as wild-type mice treated with vehicle (WT-V, n = 12) or with FA (WT-FA, n = 12) for 6 months commencing at 6 months of age. (A) Representative photomicrographs of ionized calcium-binding adapter molecule 1 (Iba1) immunohistochemistry for β-amyloid deposit-associated microgliosis are shown in PSAPP-V and PSAPP-FA mice at 12 months of age. Brain regions shown include: cingulate cortex (CC, top), hippocampus (H, middle), and entorhinal cortex (EC, bottom). Middle H panels are higher magnification images from insets in the upper H panels. Scale bars denote 250 µm (upper) and 50 µm. (B) Quantitative image analysis for Iba1 burden is shown for each brain region as indicated on the x-axis. Statistical comparisons are within brain region and between PSAPP-V and PSAPP-FA mice. (C) Expression of brain proinflammatory tumor necrosis factor-α (TNF-α) and interleukin-1β (IL-1β) cytokine mRNAs was decreased in FA-treated PSAPP mice. Data are expressed as relative fold over WT-V mice, and statistical comparisons are between PSAPP-V and PSAPP-FA mice. (D) Expression of brain oxidative stress markers [superoxide dismutase 1 (Sod1), catalase, and glutathione peroxidase 1 (Gpx1) mRNAs] was reduced in FA-treated PSAPP mice. Data are expressed as relative fold over WT-V mice, and all statistical comparisons are between PSAPP-V and PSAPP-FA mice.

Glial activation associated with β-amyloid deposits may be pathoetiologic in AD via production of numerous neurotoxic acute-phase reactants, proinflammatory cytokines, and immunostimulatory molecules [38]. Moreover, it is generally accepted that oxidative stress plays a cardinal role in the progression of AD neuropathology [39], [40]. To determine whether FA impacted neuroinflammatory processes and antioxidant activity in PSAPP mice, we examined β-amyloid deposit-associated microgliosis and astrocytosis, and then quantified brain mRNA expression of proinflammatory innate immune cytokines [tumor necrosis factor-α (TNF-α) and interleukin-1β (IL-1β)] by QRT-PCR analysis. In addition, mRNA expression of three cardinal oxidative stress markers [superoxide dismutase 1 (Sod1), catalase, and glutathione peroxidase 1 (Gpx1)] was quantified by QRT-PCR analysis. PSAPP-V mice demonstrated exacerbated β-amyloid plaque-associated reactive microgliosis and astrocytosis, as evidenced by increased expression of ionized calcium-binding adapter molecule 1 (Iba1) and glial fibrillary acidic protein (GFAP) in glial somata and processes. Numerous minute GFAP-positive granules, which were probably within astrocytic processes, were dispersed between neurons. In addition, GFAP expression was strongly detected in dystrophic neurites in association with β-amyloid deposits. It is noteworthy that PSAPP-V mice had hyperplasia and hypertrophy of activated astrocytes and microglia in and around β-amyloid deposits, which were strongly GFAP/Iba1 immunoreactive in all three brain regions examined. Both microglial and astroglial activation were significantly reduced in PSAPP-FA mice vs. PSAPP-V animals, consistent with a resting glial phenotype (Fig. 7A, B, 8A, B, **p<0.01; ***p<0.001). Supporting the notion that FA treatment mitigated neuroinflammation, brain mRNA expression of TNF-α and IL-1β was also significantly reduced in PSAPP-FA mice (Fig. 7C, *p<0.05; **p<0.01). Expression of three key oxidative stress markers (Sod1, catalase, and Gpx1) was increased in PSAPP-V mice, and expression of all three was significantly reduced in PSAPP-FA mice (Fig. 7D, *p<0.05) to the level of the control WT-FA group (p>0.05). These effects were gender-independent, as a similar pattern of statistically significant results was noted in both male and female PSAPP-FA mice (data not shown). Collectively, FA ameliorated neuroinflammatory processes and reduced expression of oxidative stress markers in PSAPP mouse brains.

**Figure 8:**
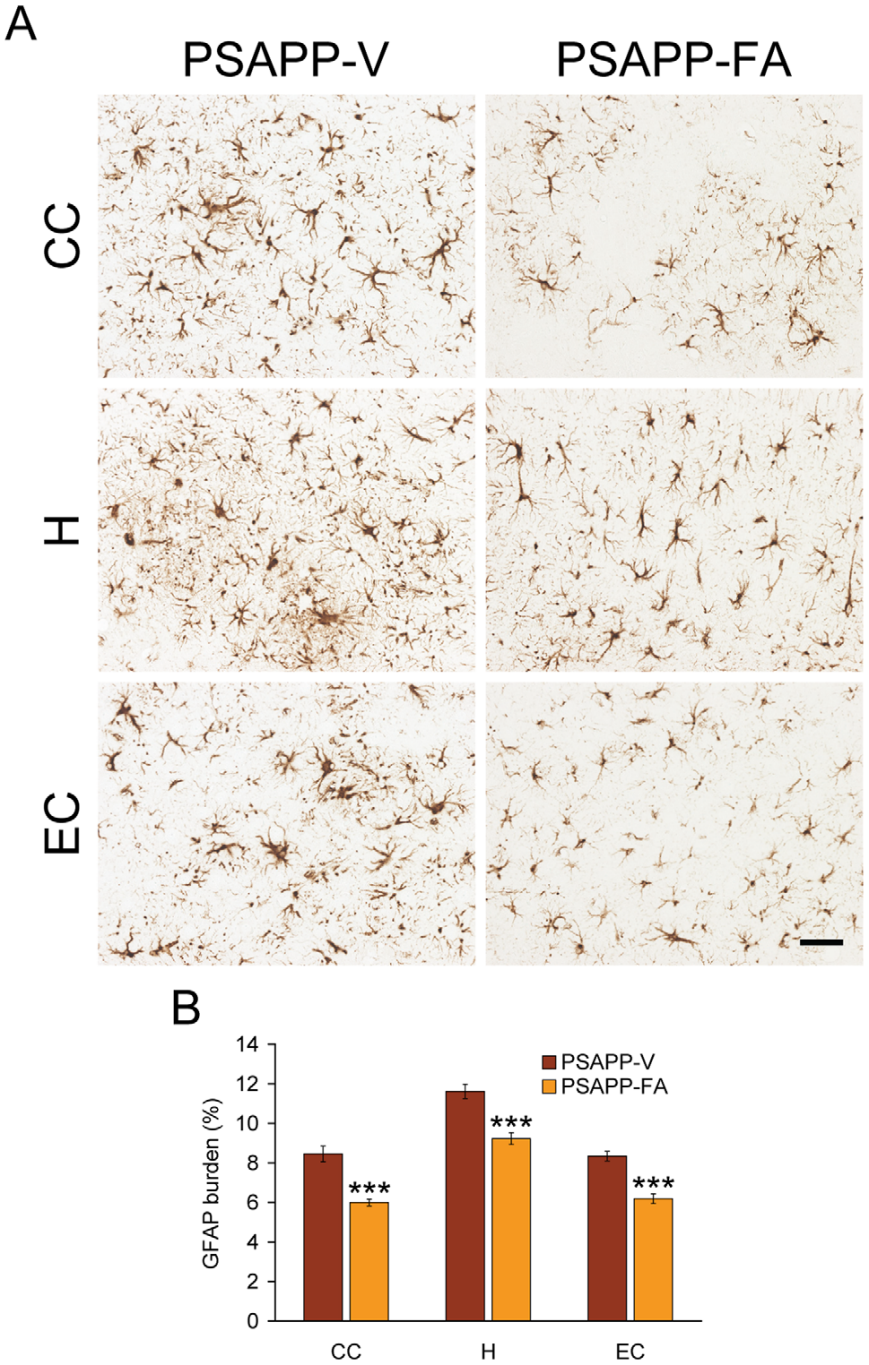
Oral treatment with ferulic acid (FA) attenuates reactive astrocytosis in PSAPP mice. Data were obtained from PSAPP mice treated with vehicle (PSAPP-V, n = 12) or with FA (PSAPP-FA, n = 12) for 6 months beginning at 6 months of age. (A) Representative photomicrographs of glial fibrillary acidic protein (GFAP) immunohistochemistry for β-amyloid deposit-associated astrocytosis are shown from PSAPP-V and PSAPP-FA mice at 12 months of age. Brain regions depicted include: cingulate cortex (CC, top), hippocampus (H, middle), and entorhinal cortex (EC, bottom). Scale bar denotes 50 µm. (B) Quantitative image analysis for GFAP burden is shown for each brain region indicated on the x-axis. All statistical comparisons are between PSAPP-V and PSAPP-FA mice.

## Discussion

In this report, we show that oral FA treatment for 6 months commencing at 6 months of age improves behavioral impairment, mitigates cerebral amyloidosis, and inhibits amyloidogenic APP metabolism by reducing BACE1 expression and β-secretase activity in PSAPP mice. Supporting results from cultured mutant human APP-overexpressing murine neuron-like cells revealed FA dose-dependent reduction of various Aβ species and inhibition of β-secretase cleavage. A cell-free BACE1 activity assay demonstrated a direct mode of FA action on inhibiting BACE1 activity. FA also ameliorated neuroinflammation in PSAPP mice including β-amyloid plaque-associated gliosis and expression of the proinflammatory cytokines, TNF-α and IL-1β. Lastly, mRNA expression of three oxidative stress markers (Sod1, catalase, and Gpx1) was decreased in FA-treated PSAPP mice. Collectively, our findings provide support for long-term FA dietary supplementation as a therapeutic strategy for AD.

Plant-derived compounds have garnered recent attention as nutraceutical treatments for a variety of diseases including neurodegenerative disorders, cancer, diabetes, and cardiovascular disease [14]. FA is one of the most abundant phenolic compounds in the human diet, and is generated in plants by the metabolism of phenylalanine and tyrosine [15]. The compound is a major constituent of many fruits and vegetables, and is found in plant seeds and leaves, both in free form and covalently linked to lignin and other biopolymers. The compound has pleiotropic biological activities including anti-inflammatory and antioxidant properties [15], [41], [42]. Given this bioactivity profile of FA, we hypothesized that long-term oral FA treatment may delay the progression of AD-like pathology, if administered early. To test this, FA was orally given to mice at 30 mg/kg/day via gavage, as this treatment strategy more precisely delivers agents compared with *ad libitum* access to drinking water or chow.

The LD_50_ of orally-administered FA is as high as 2,370 mg/kg in the mouse, and the dose that we administered to mice (30 mg/kg/day) is orders of magnitude lower. The tolerable daily intake (TDI) in humans can be extrapolated from rodent LD_50_ threshold data [43]. Assuming that the default uncertainty factor accounting for interspecies variation is 10 [44], one can calculate a TDI of 14.2 g of FA for a 60 kg human, which is well above the levels administered in this study. Of course, adverse events must be taken into account for any therapeutic agent, as such events have the potential to halt clinical trials, regardless of efficacy. Thus, it is worth noting that we did not detect any adverse events including occurrence of atypical behavior, altered food/water intake, or mortality associated with long-term FA treatment in mice. Moreover, no evidence of pathological findings in major central nervous, pleural, and abdominal organs such as the brain, lung, heart, liver, spleen, pancreas, adrenal gland, urinary bladder, kidney, and digestive tract was observed upon postmortem examination of either PSAPP or WT mice. These findings reinforce the notion that the dose of FA used in mice is safe, although toxicology analyses would need to be conducted in humans.

As to how FA exerts its biological effects, the compound is a low molecular weight molecule (194.18 g/mol). As compared with other larger phenolics, FA is freely cell-permeable and has high bioavailability in rats [15], [45]. FA is quickly absorbed in free form via stomach mucosal cells, and is transported into the hepatic portal vein where it may be conjugated with glucuronide and/or sulfate. Subsequently, remaining free and conjugated FA enters the systemic circulation and is biodistributed in the brain and peripheral tissues [15], [45]. Because the compound remains in the circulation longer than other naturally occurring compounds like vitamin C [15], FA is expected to stay in the body long enough to elicit its effects. While FA is not necessarily predicted to cross the blood-brain barrier due to it being a charged molecule with a hydroxyl group [46], others have detected the molecule in the rodent brain following peripheral administration [47]. Additionally, an ethyl ester derivative of FA has been shown to protect neurons against amyloid β-peptide-induced oxidative stress and neurotoxicity [46]. Thus, derivatives of FA may have enhanced bioactivity, and future study is warranted to examine whether FA derivatives are effective at mitigating Aβ pathology.

Our results show that FA modulates β-secretase cleavage both *in vivo* and *in vitro*. Of note, FA targets BACE1 protein stability (without affecting BACE1 mRNA expression) and β-secretase activity (but has no effect on α-secretase activity), resulting in reduced abundance of the amyloidogenic C99/P-C99 APP CTFs and Aβ species including oligomers. It deserves mentioning that FA exerts its effects on BACE1 both by limiting protein abundance and also by directly attenuating enzymatic activity, as demonstrated in a cell-free BACE1 activity assay. While inhibition of BACE1 by FA affords the opportunity to mitigate AD pathology, other BACE1 substrates such as the cell adhesion protein P-selectin glycoprotein ligand-1 [48], the APP homolog proteins APP-like protein 1/2 [49], [50], the low density lipoprotein receptor-related protein [51], the β subunit of voltage-gated sodium channels [52], and the control protein of myelination [53], [54] could be indirectly affected by modulation of BACE1 activity.

Under physiological conditions, the majority of APP molecules are processed via non-amyloidogenic α-secretase cleavage; therefore, Aβ species are constitutively generated at relatively low levels. β-secretase cleavage is thought to be a rate-limiting step for Aβ generation [33]–[37], and it is noteworthy that both α- and β-secretases compete for APP proteolysis [55]. Therefore, reducing β-secretase activity could theoretically shift the balance toward non-amyloidogenic APP processing, and this has generally been the rationale for therapeutic development of β-secretase modulators. Collectively, the data presented in this report suggest that mitigated cerebral amyloidosis in PSAPP mice and decreased Aβ secretion from SweAPP N2a cells after FA treatment are, at least partially, owed to reduced amyloidogenic APP processing.

Parenthetically, we previously demonstrated that another nutraceutical, the green tea polyphenol (–)-epigallocatechin-3-gallate (EGCG), was able to promote activity of the candidate α-secretase, a disintegrin and metalloprotease 10, thereby endorsing non-amyloidogenic APP processing in the Tg2576 cerebral amyloidosis mouse model and in SweAPP N2a cells [56], [57]. Given their complementary modes of action then, combination therapy including reduced amyloidogenic β-secretase cleavage by FA and enhanced non-amyloidogenic α-secretase cleavage by EGCG might make sense as a synergistic Aβ lowering strategy.

Newly-generated Aβ species from sequential endoproteolytic cleavage of APP by β- and γ-secretases enter into dynamic equilibrium between soluble and deposited forms in the brain, with continual transport of soluble Aβ out of the brain and into the blood [32]. We examined whether FA selectively impacted distinct pools of Aβ peptides in the brain, and found nonselective decrease in abundance of Aβ_1–40_ and Aβ_1–42_ peptides in TBS-, SDS-, and guanidine HCl-soluble brain homogenate fractions from FA-treated PSAPP mice. Recent attention has been directed toward soluble multimeric forms of Aβ as the principle toxic species. These so-called “Aβ oligomers” disrupt synaptic function and are neurotoxic *in vivo* [58]–[60]. In this regard, we found decreased abundance of oligomeric Aβ by Western blot and by sandwich ELISA in brain homogenates from FA-treated PSAPP mice. These observations may be owed to less amyloidogenic β-secretase APP proteolysis (i.e., a concentration-dependent effect on Aβ oligomer formation).

It has been shown that cerebral β-amyloid deposits do not correlate well with behavioral impairment in transgenic mice expressing APP mutations that cause early-onset familial AD [61], [62]. These findings have led others to postulate that Aβ oligomers are the primary neurotoxic species [58], [60]. Interestingly, strong correlations have been found between soluble Aβ oligomers and cognitive disturbance in mouse models of cerebral amyloidosis [62], [63]. Moreover, extracellular accumulation of a 56-kDa soluble Aβ assembly (Aβ*56) has been reported to account for memory deficits in middle-aged Tg2576 mice [64]. While selective removal of Aβ species by immunotherapeutic approaches remediates behavioral impairment in mouse models [5], [63], [65], reducing soluble Aβ alone was not sufficient to improve cognitive disturbance in 3× Tg-AD mice [66]. Thus, while Aβ is generally regarded as the pathogenic species in both mouse models of cerebral amyloidosis and human AD, it certainly remains possible that FA reduction of any combination of amyloidogenic APP metabolites could at least be partially involved in reducing behavioral impairment in PSAPP mice.

While our data suggest that FA directly reduces β-secretase activity, the compound has been reported to have other bioactivities including blocking inflammation. Neuroinflammation is an important AD pathoetiologic hallmark [38], and β-amyloid deposit-associated microgliosis and astrocytosis were reduced in FA-treated PSAPP mice, along with decreased expression of proinflammatory cytokines (TNF-α and IL-1β). One interpretation of the above results is that FA has an anti-inflammatory effect independent of its anti-amyloidogenic property, and this effect may occur at the level of the central nervous system and/or the periphery. However, neuroinflammation and cerebral amyloidosis generally correlate in human AD and in mouse models [9], [22], [29], [30], [67]–[70], and so it remains possible that reduced neuroinflammation may be secondary to decreased cerebral amyloidosis following long-term administration of FA.

It is well-accepted that oxidative stress contributes to AD neuropathology [39], [40]. For example, oxidative stress markers are elevated in neurons surrounding β-amyloid deposits in transgenic mouse models of the disease [71], and experimental induction of oxidative stress leads to Aβ accumulation in primary neurons [72]. FA possesses three structural motifs that imbue the molecule with free radical scavenging capacity (Fig. 1). For example, the presence of electron-donating groups on the benzene ring (3-methoxy and, more importantly, 4-hydroxyl) can readily form a resonance stabilized phenoxy radical. In addition, tertiary structure − the FA carboxylic acid group with adjacent unsaturated C-C double bond − can stabilize free radicals via resonance or by providing additional sites to prevent free radical membrane attack. Moreover, this carboxylic acid group can act as a lipid anchor, thereby affording protection against lipid peroxidation [73]. Given the antioxidant property of FA, we explored whether the compound might modulate oxidative stress in PSAPP mice. Importantly, long-term oral FA administration reduced cerebral expression levels of three key oxidative stress markers (Sod1, catalase, and Gpx1) to baseline levels.

In sum, data presented in this report reinforce the notion that the naturally occurring dietary compound, FA, remediates behavioral impairment, reduces amyloidogenic APP metabolism by modulating β-secretase, and mitigates AD-like pathology in the PSAPP transgenic mouse. If cerebral amyloid pathology in this transgenic mouse model is representative of the clinical syndrome, then long-term FA treatment could prove to be a safe and effective disease-modifying therapeutic.

## Materials and Methods

### Ethics Statement

All experiments were performed in accordance with the guidelines of the NIH, and all animal studies were approved by the Saitama Medical University Institutional Animal Care and Use Committee. Animals were humanely cared for during all experiments, and all efforts were made to minimize animal suffering. Animals were anesthetized with sodium pentobarbital (50 mg/kg) and euthanized by transcardial perfusion with ice-cold physiological saline containing heparin (10 units/ml).

### Mice

Male B6.Cg-Tg(APPswe, PSEN1dE9)85Dbo/Mmjax mice (bearing APPswe and PS1_ΔE9_ mutant human transgenes) on the congenic C57BL/6J background (designated PSAPP mice) were obtained from the Jackson Laboratory (Bar Harbor, ME) and were bred with female C57BL/6J mice to yield PSAPP and WT offspring. PSAPP mice overproduce human Aβ_1–40_ and Aβ_1–42_ peptides and develop progressive cerebral β-amyloid deposits and learning and memory impairment [21], [22], [27], [28], [74]. All mice were characterized by PCR genotyping for mutant human APP and PS1 transgenes as described elsewhere [74]. We strictly used PSAPP and WT littermates obtained from this breeding strategy for all analyses. Thus, all mice used in this study are genetically comparable.

FA was obtained from Sigma-Aldrich (St. Louis, MO), resuspended in distilled water, and orally administered to 12 PSAPP mice (PSAPP-FA mice; 6 males and 6 females). As a vehicle control, 12 additional PSAPP mice received distilled water (PSAPP-V mice; 6 males and 6 females). In addition, 24 WT littermates received FA (WT-FA mice; 6 males and 6 females) or distilled water (WT-V mice, 6 males and 6 females). Baseline cognitive status was determined in untreated PSAPP vs. WT mice at 6 months of age just prior to dosing. Subsequently, animals were gavaged with FA (30 mg/kg) or vehicle once daily for 6 months. Mice were housed in a specific pathogen-free barrier facility under a 12/12-hour light-dark cycle, with *ad libitum* access to food and water.

### Behavioral Analyses

Exploratory activity was evaluated by individually placing mice into a novel environment (the left corner of a white polyethylene chamber; 54×39×20 cm). Their activity was recorded for 20 minutes by an overhead video camera (BL-C131, Panasonic, Fukuoka, Japan) connected to a Windows PC, and horizontal locomotion and rearing scores were counted for each 2-minute time bin [23], [75].

Novel-object recognition and memory retention were assessed as described [24]. Briefly, each mouse was habituated in a cage for 4 hours, and then two objects of different shapes were concurrently provided to the mouse for 10 minutes. The number of times that the mouse explored the object (defined as number of instances where a mouse directed its nose 2 cm or less distance from the object) that was later replaced by a novel object were counted for the initial 5 minutes of exposure (training phase). To test memory retention on the following day, one of the original objects was replaced with a different shaped novel object, and then the number of explorations of the novel object was counted for 5 minutes (retention test). The recognition index, taken as an index of memory, is reported as frequency (%) of explorations of the novel vs. original objects.

Subsequently, Y-maze total arm entry and spontaneous alternation were assessed to measure exploratory activity and spatial working memory [9], [21]. Briefly, mice were individually placed in one arm of a radially symmetric Y-maze made of opaque gray acrylic (arms: 40 cm long, 4 cm wide; walls: 30 cm tall), and the sequence of arm entries and total number of entries were recorded over a period of 8 minutes, beginning when the animal first entered the central area. Per cent alternation was defined as entries into sequentially different arms on consecutive occasions using the following formula: % alternation = Number of alternations/(Number of total arm entries minus 2)×100.

Lastly, Morris water maze testing was performed essentially as previously described [25], [26] to assess spatial reference learning and memory. The water maze consisted of a circular pool (80 cm diameter) filled with water maintained at 23 to 26°C. For the purpose of *post hoc* analyses, the pool was divided into four quadrants (Q 1 to Q 4), and a 6-cm diameter plexiglass platform was located 1 cm above the water surface in the center of Q 2. After a minimum of 20 minutes habituation to the room, mice naïve to the test were placed in the pool and allowed to search for the platform for 60 seconds. On the first 2 days (four trials were conducted per day with a 20-minute inter-trial interval), a visible cue was placed on the platform and its location was randomly varied amongst four possible locations (counterbalanced across mice). The trial ended when a mouse climbed the platform, or in the allocated 60 seconds, whichever came first. After finding and climbing on the platform, each mouse was allowed to remain there for 20 seconds, and was then returned to its cage. Animals that did not locate the platform within 60 seconds were guided to it and allowed to remain there for 20 seconds before being returned to their cages. On the third day, submerged platform testing was conducted for five consecutive days (learning phase; four trials per day with a 20-minute inter-trial interval). The location of the indiscernible platform remained in Q 2, 1 cm below the water surface, and mice were placed into the pool in one of seven randomly selected locations (excluding the position immediately adjacent to the platform). One day after the conclusion of the learning phase, memory retention was determined in a single 60-second probe trial. The submerged platform was removed from the water maze, and mice were placed and released opposite the site where the platform had been located and time spent in each quadrant was recorded for the probe trial.

All behavioral tests were performed in a room (6 m×4.5 m) with indirect lighting and multiple visible cues on the walls. The examiner determined the time of swimming until the mouse reached the platform (latency) using a stopwatch. In addition, trials were recorded using an overhead video camera and were analyzed using customized macro software in Microsoft Excel. All trials were performed at the same time of day (±1 hour), during the animals’ light phase. So as not to interfere with behavioral testing, FA or vehicle treatment was carried out 1 hour after concluding behavioral testing.

### Tissue Preparation

Tissue was processed according to our previously described methods [7], [9], [22], [29], [30]. At 12 months of age, animals were anesthetized with sodium pentobarbital (50 mg/kg) and euthanized by transcardial perfusion with ice-cold physiological saline containing heparin (10 units/ml). Brains were isolated and quartered (sagittally at the level of the longitudinal fissure of the cerebrum, and then coronally at the level of the anterior commissure) using a mouse brain slicer (Muromachi Kikai, Tokyo, Japan). Right anterior cerebral quarters were weighed and snap-frozen at −80°C for α- or β-secretase activity analyses. Right posterior cerebral quarters were further divided into two pieces, and weighed and snap-frozen at −80°C. One-half was sequentially extracted in 1) TBS (25 mM Tris-HCl, pH 7.4, 150 mM NaCl), 2) 2% SDS, and 3) 5 M guanidine-HCl for sandwich ELISAs. The other half was homogenized and used for Western blot analyses. Left anterior cerebral quarters were weighed and immersed in RNA stabilization solution (RNAlater®, Applied Biosystems, Foster City, CA) and then snap-frozen at −80°C for QRT-PCR analyses. Left posterior cerebral quarters were immersion fixed in 4% paraformaldehyde in 0.1 M phosphate buffer at 4°C overnight, and routinely processed in paraffin for immunohistochemical analyses.

### Immunohistochemistry

For paraffin blocks, we sectioned five coronal sections (per set) with a 100-µm interval and a thickness of 5-µm for each brain region (for CC, bregma −0.10 to −0.82 mm; for H and EC, bregma −2.92 to −3.64 mm) [76]. Three sets of five sections were prepared for each brain region for analyses of Aβ deposits/β-amyloid plaques (for burden and plaque morphometry analyses) as well as Iba1 (a reactive microglia marker) and GFAP (an astrocytosis marker) burdens. Immunohistochemical staining was conducted according to the manufacturer’s protocol using a Vectastain ABC *Elite* kit (Vector Laboratories, Burlingame, CA) coupled with the diaminobenzidine reaction, except that the biotinylated secondary antibody step was omitted for Aβ immunohistochemical staining. The following primary antibodies were variously used: a biotinylated human Aβ_17–24_ monoclonal antibody (4G8; 1∶200, Covance Research Products, Emeryville, CA), an Iba1 polyclonal antibody (1∶1,000, Wako, Osaka, Japan), and a polyclonal GFAP antibody (1∶500, Dako, Carpinteria, CA). Using additional sets of five sections, normal mouse or rabbit serum (isotype control) or 0.1 M PBS (pH 7.4) was used instead of primary or secondary antibody or ABC reagent as a negative control.

### Image Analysis

Quantitative image analysis was done based on previously validated methods [7], [9], [22], [29], [30]. Images were acquired as digitized tagged-image format files to retain maximum resolution using a BX60 microscope with an attached CCD camera system (DP-70, Olympus, Tokyo, Japan), and digital images were routed into a Windows PC for quantitative analyses using SimplePCI software (Hamamatsu Photonics, Hamamatsu, Shizuoka, Japan). We captured images of five 5-µm sections through each anatomic region of interest (CC, EC, and H) based on anatomical criteria defined by Franklin and Paxinos [76], and obtained a threshold optical density that discriminated staining from background. Each anatomic region of interest was manually edited to eliminate artifacts. For Aβ, Iba1 (microgliosis), and GFAP (astrocytosis) burden analyses, data are reported as the percentage of labeled area captured (positive pixels) divided by the full area captured (total pixels). Selection bias was controlled for by analyzing each region of interest in its entirety.

For β-amyloid plaque morphometric analyses, diameters (based on maximum length) of β-amyloid plaques were measured, and numbers of β-amyloid plaques falling into three mutually exclusive diameter categories (<25, 25–50, or >50 µm) were tabulated. Results are presented as mean plaque number per mouse for each brain region surveyed. For CAA morphometric analysis, we counted numbers of Aβ antibody-stained cerebral vessels in each anatomic region of interest based on our previous methods [22], [30]; those data are represented as mean CAA deposit numbers per mouse.

### Cell Culture

SweAPP N2a cells were kindly provided by Dr. Gopal Thinakaran (Department of Neurobiology, University of Chicago) [77]. SweAPP N2a cells were grown in Dulbecco’s modified Eagle’s medium supplemented with 10% fetal calf serum, 2 mM glutamine, 100 units/ml of penicillin, 0.1 µg/ml of streptomycin, and 200 µg/ml of G418 sulfate according to previously described methods [7], [22], [56], [78]. SweAPP N2a cells were seeded in 24-well tissue culture plates at 1×10^5^ cells per well. Cultured cells were differentiated into neuron-like cells by 2 hours pre-treatment with neurobasal media containing 300 µM dibutyryl cAMP and then treated with FA (1.563, 3.125, 6.25, or 12.5 µM) or 0.1 M PBS (pH 7.4; control) for 12 hours in the same media prior to analysis.

### Lactate Dehydrogenase Release Assay

SweAPP N2a cells were seeded in 24-well tissue culture plates at 1×10^5^ cells per well. Cultured cells were differentiated into neuron-like cells by 2 hours pre-treatment with neurobasal media containing 300 µM dibutyryl cAMP and then treated with FA (1.563, 3.125, 6.25, or 12.5 µM) or 0.1 M PBS (pH 7.4; control) for 12 hours in the same media. Culture wells were then assayed for cell death by a lactate dehydrogenase release assay (Promega, Madison, WI) as described [22], [79].

### Cell-free BACE1 Activity Assay

To directly test the effect of FA on BACE1 activity, we used available kits based on secretase-specific peptides conjugated to DABCYL/EDANS fluorogenic reporter molecules (Cayman Chemical, Ann Arbor, MI) in accordance with the manufacturer’s instructions and our previously described methods [22]. Briefly, BACE1 enzyme was incubated with various concentrations of FA (1.563, 3.125, 6.25, or 12.5 µM) or BACE1 inhibitor II (1.25 µM, as a positive control; Merck Millipore, Darmstadt, Germany) in the presence of 1×reaction buffer for 40 minutes prior to reading fluorescence values on a FLUOstar Omega (BMG LABTECH, San Diego, CA) fluorescent microplate reader.

### ELISA

We separately quantified Aβ_1–40_ and Aβ_1–42_ in brain homogenates and cultured SweAPP N2a cell supernatants by sandwich ELISAs. Brain Aβ_1–40_ and Aβ_1–42_ species were detected by a three-step extraction protocol according to previously published methods [80], [81]. Briefly, brains were homogenized using TissueLyser LT (Qiagen, Valencia, CA; two times for 1 minute at 50 Hz) in TBS solution containing protease inhibitor cocktail (Sigma-Aldrich), centrifuged at 18,800×g for 60 minutes at 4°C, and supernatants were removed (representing the TBS-soluble fraction). Resulting pellets were treated with 2% SDS in H_2_O with the same protease inhibitors and homogenized using TissueLyser LT (one time for 1 minute at 50 Hz). We then centrifuged the homogenates at 18,800×g for 60 minutes at 4°C and collected supernatants (comprising the 2% SDS-soluble fraction). Finally, remaining pellets were treated with 5 M guanidine HCl and dissolved by occasional mixing on ice for 30 minutes, centrifuged at 18,800×g for 60 minutes at 4°C, and supernatants were then collected representing the guanidine HCl-soluble fraction.

Aβ_1–40_ and Aβ_1–42_ species were separately quantified in individual samples in duplicate using ELISA kits (catalogue number 27718 for Aβ_1–40_ and number 27712 for Aβ_1–42_; IBL, Fujioka, Gunma, Japan) in accordance with the manufacturer’s instructions [82]. We also quantified Aβ oligomers in the 2% SDS-soluble fraction in duplicate individual samples by Aβ oligomer ELISA (catalogue number 27725; IBL) according to the manufacturer’s instructions [83]. All samples fell within the linear range of the standard curve. For brain homogenates, Aβ_1–40_ and Aβ_1–42_ ELISA values are reported as picomolar or nanomolar, and Aβ oligomer concentration is reported as picomolar. For cultured SweAPP N2a cell supernatants, Aβ_1–40_ and Aβ_1–42_ ELISA values are reported as picograms of Aβ_1-X_/mg of cellular protein.

### Western Blot

Cultured SweAPP N2a cells were treated with various doses of FA (1.563, 3.125, 6.25, or 12.5 µM) or 0.1 M PBS (pH 7.4; control) for 12 hours. Cultured cells were then lysed in ice-cold lysis buffer (containing 20 mM Tris-HCl pH 7.5, 150 mM NaCl, 1 mM Na_2_EDTA, 1 mM EGTA, 1% Triton X-100, 2.5 mM sodium pyrophosphate, 1 mM β-glycerophosphate, 1 mM Na_3_VO_4_, 1 µg/ml leupeptin, and 1 mM PMSF). Mouse brain homogenates were lysed in TBS solution containing protease inhibitor cocktail (Sigma-Aldrich) followed by TNE buffer (10 mM Tris-HCl, 1% NP-40, 1 mM EDTA, and 150 mM NaCl), and aliquots corresponding to 10 µg of total protein were electrophoretically separated using 10 or 15% Tris glycine gels based on target protein molecular weights. Electrophoresed proteins were transferred to polyvinylidene difluoride membranes (Bio-Rad, Richmond, CA) that were blocked in blocking buffer [1% (w/v) nonfat dry milk in TBS containing 0.1% (v/v) Tween-20] for 1 hour at ambient temperature. Membranes were then hybridized for 1 hour at ambient temperature with primary antibodies: an amino-terminal APP polyclonal antibody (1∶400, IBL), a carboxyl-terminal APP polyclonal antibody (1∶500, Merck Millipore), a carboxyl-terminal PS1 monoclonal antibody (PS1-loop; 1∶500, Merck Millipore), a carboxyl-terminal BACE1 polyclonal antibody (1∶400, IBL), an amino-terminal Aβ_1–16_ monoclonal antibody (82E1; 1∶150, IBL), an amino-terminal Aβ_1–17_ monoclonal antibody (6E10; 1∶1,000, Merck Millipore), or a polyclonal actin antibody as a loading control (1∶500, Santa Cruz Biotechnology, Santa Cruz, CA). Membranes were then rinsed three times for 30 minutes each in TBS containing 0.1% (v/v) Tween-20 and incubated for 1 hour at ambient temperature with appropriate horseradish peroxidase-conjugated secondary antibodies. After additional rinsing as above, membranes were incubated for 5 minutes at ambient temperature with enhanced chemiluminescence substrate (SuperSignal West Femto Extended Duration Substrate, Thermo Fisher Scientific, Waltham, MA), exposed to film, and developed.

### Secretase Activity Assays

For α- and β-secretase activity analyses in brain homogenates, we used available kits based on secretase-specific peptides conjugated to fluorogenic reporter molecules (DABCYL/EDANS; R & D Systems, Minneapolis, MN) according to our published methods [22], [30], [56]. Briefly, brains were lysed in ice-cold 1× cell extraction buffer for 10 minutes and centrifuged at 18,800×g for 1 minute. Supernatants were collected and kept on ice. Appropriate amounts of brain homogenate, reaction buffer, and fluorogenic substrate were added in duplicate to a 96-well plate and incubated in the dark at 37°C. Following incubation, fluorescence (335 nm excitation and 495 nm emission) was monitored at 25°C using a SH-9000 microplate fluorimeter with SF6 software (CORONA ELECTRIC, Hitachinaka, Ibaraki, Japan). Background was determined from negative controls (omission of brain homogenate or fluorogenic substrate).

### QRT-PCR

We quantified TNF-α, IL-1β, BACE1, Sod1, catalase, Gpx1, and β-actin mRNA levels by QRT-PCR analysis. Total RNA was extracted using RNeasy Mini Kit (Qiagen), and first strand cDNA synthesis was carried out using the QuantiTect Reverse Transcription Kit (Qiagen) in accordance with the manufacturer’s instructions. We diluted cDNA 1∶1 in H_2_O and carried out QRT-PCR for all genes of interest using cDNA-specific TaqMan primer/probe sets (TaqMan Gene Expression Assays, Applied Biosystems) on an ABI 7500 Fast Real-time PCR instrument (Applied Biosystems). Each 20-µl reaction mixture contained 2 µl of cDNA with 1 µl of TaqMan Gene Expression Assay reagent, 10 µl of TaqMan Fast Universal PCR Master Mix (Applied Biosystems), and 7 µl of H_2_O. Thermocycler conditions consisted of: 95°C for 15 seconds, followed by 40 cycles of 95°C for 1 second and 60°C for 20 seconds. TaqMan probe/primer sets were as follows: mouse TNF-α (catalogue number Mm00443258_m1), mouse IL-1β (number Mm00434228_m1), mouse BACE1 (number Mm00478664_m1), mouse Sod1 (number Mm01700393_g1), mouse catalase (number Mm00437992_m1), mouse Gpx1 (number Mm00656767_g1), mouse β-actin (number Mm00607939_s1; used as an internal reference control), and Rps29 (number Mm02342448_gH; used as an internal reference control) (Applied Biosystems). Samples that were not subjected to reverse transcription were run in parallel as negative controls to rule out genomic DNA contamination, and a “no template control” was also included for each primer set (data not shown). The cycle threshold number (*C*_T_) method [84] was used to determine relative amounts of initial target cDNA in each sample. Results are expressed relative to vehicle-treated WT mice. The levels of BACE1 mRNA extracted from SweAPP N2a cells were normalized with RNA levels of Rsp29 (a housekeeping gene).

### Statistical Analysis

All experiments were performed by an examiner blinded to sample or subject identities, and code was not broken until the analyses were completed. Data are presented as the mean ±1 S.E. A hierarchical analysis strategy was used for time-dependent behavioral data in which the first step was a repeated-measures ANOVA to assess the significance of the main effects and interactive terms. If significant, *post hoc* testing was done with Tukey’s HSD or Dunnett’s T3 methods, and appropriate p values are reported based on adjustment according to Levene’s test for equality of the variance. For all other data, in instances of single mean comparisons, Levene’s test followed by t test for independent samples was performed. In instances of multiple mean comparisons, one-way ANOVA was used, followed by *post hoc* comparison of the means using Bonferroni’s or Dunnett’s T3 methods (where appropriateness was determined by Levene’s test for equality of the variance). A p value of less than 0.05 was considered to be significant. All analyses were performed using the Statistical Package for the Social Sciences, release IBM SPSS 19.0 (IBM, Armonk, NY).
